# Isolation, functional evaluation, and fermentation process optimization of probiotic *Bacillus coagulans*

**DOI:** 10.1371/journal.pone.0286944

**Published:** 2023-11-03

**Authors:** Li Xu, Zhi Chun Zhan, ShiShen Du, ShaoYun Wang, QianQian Zhang, Chao Wang, WenJie Yang, XiaoXu Deng, ZeTao Zhan, Yang Li, Ying Zhou, XiangDong Chen

**Affiliations:** 1 State Key Laboratory of Virology, College of Life Sciences, Wuhan University, Wuhan, P.R. China; 2 Wuhan SunHY Biology Co., Ltd., Wuhan, P.R. China; 3 Sunhy Technology (Hubei) Co., Ltd., Huanggang, P.R. China; 4 China Center for Type Culture Collection, Wuhan, P.R. China; Tanta University Faculty of Agriculture, EGYPT

## Abstract

*Bacillus coagulans* is a probiotic agent widely used in various industries. In this study, we isolated a novel strain of *B*. *coagulans*, X26, from soil and characterized its properties. X26 exhibited superior enzyme, acid, and biomass yields when compared with other bacterial probiotics and an antibiotic. Moreover, X26 significantly improved the body weight of rats, highlighting its potential for industrial development as a supplement for animals. To optimize the fermentation process of this bacterium, we adopted the response surface design. When X26 was cultured in a medium with 16.5 g/L maltose, 25.00 g/L yeast extract, and 3.5 g/L K_2_HPO_4_, the optimal yield was predicted to be 5.1 × 10^9^ CFU/mL. Consistent with the prediction, the yield of X26 in a 500-mL flask culture was (5.12 ± 0.01) × 10^9^ CFU/mL, and in a 30-L fermenter was (5.11 ± 0.02) × 10^9^ CFU/mL, accounting for a 9.9-fold higher field than that with a basal medium before optimization. We further optimized the fermentation process in the 30-L and a 10-T fermenter, generating yields of (7.8 ± 0.2) × 10^9^ CFU/mL (spore rate: 96.54%) and (8.7 ± 0.1) × 10^9^ CFU/mL (spore rate: 97.93%), respectively. These yields and spore rates were achieved at 45–55°C, the typical fermentation temperature of *B*. *coagulans*. Our findings indicate that *B*. *coagulans* X26 is a promising probiotic with considerable potential for cost-effective industrial fermentation.

## Introduction

In recent years, probiotics have attracted extensive research interest for applications in several industries, including medicine, food, agriculture, animal husbandry, and fisheries. *Bacillus coagulans* is a facultative anaerobic bacterium that can adapt to low-oxygen intestinal conditions and ferment lactic acid to produce L-lactic acid, which lowers intestinal pH and inhibits the proliferation of harmful bacteria. As such, *B*. *coagulans* also serves as a probiotic. Indeed, Zhou et al. introduced the application of *B*. *coagulans* in animal husbandry [1], whereas Mu and Cong highlighted the importance of *B*. *coagulans* in medicine and its therapeutic effects for acute diarrhea, irritable bowel syndrome, antibiotic-associated diarrhea, constipation, and colitis [2]. Konuray et al. further revealed the widespread application of *B*. *coagulans* in the food industry as a probiotic [3]. Accordingly, *B*. *coagulans* is considered to be a safe and effective probiotic.

Although the beneficial effects of *B*. *coagulans* have been confirmed in different industries, its application remains limited owing to its relatively low fermentation yield, high cost, lack of serial research on the fermentation process, and discrepancies between the reported liquid fermentation temperature and biomass. At a fermentation temperature of 30–40°C, the reported number of bacterial cells in the fermentation broth is 4.5 × 10^9^–6.17 × 10^11^ CFU/mL, and the number of spores is 8.4 × 10^8^–5.74 × 10^11^ CFU/mL [4–13]. However, among the previous studies on *B*. *coagulans* fermentation, Yin et al. [4–6, 9, 10, 13] did not consider the effect of different temperatures on the yield, whereas Sun et al. [7, 11, 12] only evaluated the effect of temperature after optimization. Additionally, strain identification has rarely been performed [6–10, 12, 13].

The growth temperature of *B*. *coagulans* ranges from 30–60°C; however, the optimal growth temperature is 45–55°C, producing a yield of 1.15 × 10^9^–3.2 × 10^12^ CFU/mL and a spore number of 3.38 × 10^8^–4.57 × 10^9^ CFU/mL [14–17]. The spore yield obtained by fermentation at the typical *B*. *coagulans* growth temperature is significantly lower than that at a fermentation temperature of 30–40°C. Hence, several factors limit the practical application of *B*. *coagulans*, especially its characteristics and performance under different fermentation conditions. Therefore, further screening of high-yield strains and optimization based on the biological characteristics of *B*. *coagulans* is needed to promote the industrialized production of *B*. *coagulans* under 45–55°C culture conditions.

In this study, we aim to selectively isolate and screen *B*. *coagulans* strains with high levels of stress resistance from soil and establish an effective fermentation process and industrial development route to ultimately promote its commercial application. The findings of this study, specifically related to cultivation conditions, provide a useful reference for research in the fields of probiotic development and application.

## Materials and methods

### Selective isolation and screening of *B*. *coagulans*

Fourteen mixed acid soils were collected from orchards and vegetable fields in different regions (S1 Table); 3.0 g of each was weighed, mixed in 100 mL of sterile saline, shaken in a water bath at 37°C and 150 rpm, and extracted for 1 h. The extract was treated in a water bath at 80°C for 10 min, and the 5% inoculum was treated in a pH 2.0 yeast extract peptone-dextrose (YPD) liquid medium containing 0.3% porcine bile salts and 0.1 mg/mL 7012 pepsin (Sigma-Aldrich, St. Louis, MO, USA) at 37°C for 2 h. Subsequently, the extract was pressed, transferred to a YPD liquid medium for enrichment culture over 18 h, and then diluted and spread in a Man–Rogosa–Sharpe (MRS) medium plate (pH 5.5) for isolation and purification. Colonies were selected for identification after culturing at 55°C for 48 h.

16S rDNA was used for preliminary identification during the screening process. No further identification of non-target strains was performed. *B*. *coagulans* was first identified using 16S rDNA, and *B*. *coagulans* X26 was sent to the China Type Culture Collection Center—an International Depository Authority (IDA) approved by the World Intellectual Property Organization (WIPO)—for preservation and identification based on its appearance, physiology, biochemistry, and molecular markers. The deposit number of *B*. *coagulans* X26 is CCTCC-JD-2023061.

### Soil collection statement

Currently, no regulated protocols exist for the sampling of soil from general locations, such as farmlands, in China. The soil samples used in this study were collected from vegetable gardens or orchards, and approximately only 100 g of each soil sample was required. Therefore, only verbal consent was obtained from the landowner for the collection of samples.

### Growth and metabolic performance

A single *B*. *coagulans* X26 colony was transferred to an MRS medium and cultivated at a constant temperature of 45°C for 24–48 h. The fermentation mixture was collected for lactic acid detection by high-performance liquid chromatography (HPLC). Xylanase production was determined using xylan as the carbon source as previously described [18]. *α*-Galactosidase production was determined as previously described [19].

### *Bacillus coagulans* spore detection

The bacterial solution was treated in a water bath at 80°C for 10 min as the spore treatment condition, and viable bacteria were counted on an MRS medium plate after incubation at 45°C for 36 h.

### Rat maintenance and treatments

Rats were bred and maintained as previously described [20], with certain modifications. A total of 60 female Sprague-Dawley (SD) rats (8-week-old) were maintained at 22 ± 2°C and 45–60% humidity, under a 12-h light/dark cycle, with adaptive feeding for 1 week and breeding for 3 weeks. Each animal was raised in a single cage. The rats were randomly divided into five groups. The treatment groups received 100 g/t oxytetracycline, 1.0 × 10^6^ CFU/g *B*. *coagulans* X26, 1.0 × 10^6^ CFU/g *B*. *licheniformis*, or 1.0 × 10^6^ CFU/g *Enterococcus faecium*, respectively. The blank control rats received compound feed (Wuhan Spring Dragon Laboratory Animal Feed, Wuhan, China) comprising wheat, corn, soybean meal, fish meal, bran, soybean oil, yeast, calcium hydrogen phosphate, calcium bile acid, lysine, methionine, multidimensional and multi-minerals. The guaranteed value of feed components (contained in each kilogram of feed) was as follows: moisture ≤ 10%, crude protein ≥ 20%, crude fat ≥ 4%, crude fiber ≤ 5%, crude ash ≤ 8%, calcium 1.0–1.8, phosphorus 0.6–1.2, calcium: phosphorus 1.2:1–1.7:1, lysine ≥ 1.32, and methionine ≥ 0.78. The rats were provided with 30 g of feed and water *ad libitum* daily. Rats were weighed before feeding and every 7 d after feeding. Adverse symptoms, such as diarrhea, were routinely monitored.

### Ethics statement

This study was approved by the Experimental Animal Management and Use Committee of the Hubei Provincial Center for Disease Control and Prevention (project approval number 202120134).

Euthanasia was performed by cervical dislocation after anesthesia via intraperitoneal injection of 1% barbital sodium.

### Liquid fermentation process

#### Shake flask culture

The glycerol tube was streaked on an MRS medium slant and cultured at 45°C for 48 h. Single colonies were transferred to an MRS liquid medium and cultured on a shaker at 45°C and 180 rpm for 8–10 h to form a seed solution. Next, 5 mL of a seed solution was inoculated into a 500-mL shake flask (loading volume 100 mL) and cultured at 45°C and 180 rpm for 24–48 h.

#### 30-L fermentation tank process

We inoculated 100 mL of a seed solution into a 30-L tank (loading capacity 15 L) under the following culture conditions: 45°C, pH 7.0, tank pressure 0.035–0.05 MPa, aeration ratio 1.0 m^3^/(m^3^·min), stirring speed 100 rpm, and culture period 24–48 h. Foaming was controlled by the flow of silicone oil 1520. After feeding with 2 L of glucose and 2 L of yeast extract that was sterilized at 118°C for 30 min and administered at a feed rate of 260 mL/h, fermentation conditions were regulated at a ventilation ratio of 1.6 m^3^/(m^3^·min), stirring speed of 200–400 rpm, and a dissolved oxygen rate between 10% and 50% which was maintained by adjusting the air volume and speed.

#### 10-T production tank fermentation process

A total of 300 mL of seed solution was placed in a 500-L seed tank (loading capacity of 300 L) at a culture temperature of 45°C, pH 7.0, tank pressure 0.03–0.05 MPa, aeration ratio 1.0 m^3^/(m^3^·min), stirring speed 50–80 rpm, and culture period 8–10 h, and then transferred to a 10-T fermentation tank (loading capacity 6 m^3^). The tank prefeeding process was conducted at 45°C, pH 7.0, tank pressure 0.03–0.05 MPa, aeration ratio 1.0 m^3^/(m^3^·min), and stirring speed 50–100 rpm. After feeding with 800 L of glucose and 800 L of yeast extract at a feeding speed of 104 L/h, fermentation conditions were regulated at a stirring speed of 100–150 rpm, ventilation ratio of 1.6 m^3^/(m^3^·min), and a dissolved oxygen rate between 10% and 50% which was maintained by adjusting the air volume and speed.

#### Preparation of live bacteria

The fermentation broth was cooled with a solid content of approximately 4% to 25°C, concentrated approximately five times with a disc centrifuge, treated with 15% starch and 5% trehalose as a carrier, mixed well, and spray-dried (inlet air temperature: 160–185°C; outlet air temperature: 65–85°C) while stirring to prepare powdered *B*. *coagulans* X26.

### Test design

#### Single-factor experiment

The basal medium (control treatment) comprised glucose 10 g/L, soybean meal powder 15 g/L, KH_2_PO_4_ 5 g/L, MgSO_4_·7H_2_O 0.2 g/L, and MnSO_4_ 1.0 g/L, at pH 7.0–7.2 and was sterilized at 115°C for 30 min. To the basal medium, 10 g/L glucose, 15 g/L fructose, 15 g/L sucrose, 15 g/L maltose, 10 g/L lactose, 15 g/L maltodextrin, 15 g/L cornstarch, and 15 g/L bran flour were added as carbon sources for screening and comparison; 20 g/L soybean meal powder, 30 g/L corn steep liquor, 20 g/L yeast extract, 20 g/L peptone, 30 g/L corn gluten meal, 20 g/L corn peptone, soybean 20 g/L peptone, and 20 g/L yeast autolyzed powder were added as nitrogen sources for screening and comparison; and 0.2 g/L magnesium sulfate, 1.0 g/L manganese sulfate, 0.2 g/L calcium chloride, 3.0 g/L calcium carbonate, 3.0 g/L ammonium sulfate, 3.0 g/L dipotassium hydrogen phosphate, 3.0 g/L potassium dihydrogen phosphate, 3.0 g/L sodium chloride, 3.0 g/L sodium acetate trihydrate, 1.0 g/L L-glutamic acid, and 0.5 g/L cysteamine hydrochloride were added as inorganic salts or trace elements for comparison.

#### Plackett–Burman (PB) design

In the single-factor experiment, nine variables with a marked impact on *B*. *coagulans* X26 yield were screened and included as key factors in the PB design, including three carbon sources (lactose, glucose, and maltose), corn steep liquor, corn gluten meal, yeast extract, three nitrogen sources, manganese sulfate, dipotassium hydrogen phosphate, glutamic acid, and three trace elements. The PB design 0 level was determined according to the results of the single-factor experiment, and the high and low levels were set 1.25–2 times higher and lower than the 0 level, respectively.

#### Steepest slope test

Based on the PB design results, we optimized the horizontal concentration of the three most important variables to design the steepest slope test. The search direction was determined by the linear coefficient of the equation. According to the previous single-factor experiment, the increased step size was set as maltose 1.5 g/L, yeast extract 5.00 g/L, and K_2_HPO_4_ 0.5 g/L; other variables were at the center point level.

#### Center combination design (CCD)

According to the PB design and the results of the steepest slope test, the CCD design was carried out with 16.5 g/L maltose, 25.00 g/L yeast extract, and 3.5 g/L K_2_HPO_4_ as the center points at five levels (–1.68179, –1, 0, +1, and +1.68179).

### Statistical analysis

Design Expert 11 software (Stat-Ease, Minneapolis, MN, USA) was used in the experimental design and for statistical analysis. The fit of model equations was determined using the coefficient of determination (R^2^), the statistical significance of differences between groups was determined using Fisher’s F test, and the significance of the regression coefficient was determined using Student’s *t*-tests. The statistical significance of the data was judged by the *p*-value (< 0.05).

## Results

### Isolation and screening of *B*. *coagulans*

To isolate novel *B*. *coagulans* strains with probiotic potential, we inoculated soil sample extracts on media at different culture temperatures and stress conditions. After screening 14 soil samples, two *B*. *coagulans* strains were isolated in the quadruple stress resistance test group incubated at 55°C. Strain *B*. *coagulans* X26 was isolated from the MRS5.5 medium, whereas strain *B*. *coagulans* X60 was obtained from the nutrient agar (NA) medium. At 37°C, no *B*. *coagulans* strains were isolated from any test group; bacterial species isolated under these conditions were primarily *B*. *cereus* [21], *B*. *siamese*, *B*. *amyloliquefaciens*, *B*. *veles*, *B*. *subtilis*, *B*. *chiella*, *B*. *licheniformis*, *B*. *paralicheniformis*, and *B*. *desert Sonora*. *B*. *stuarteri* was obtained from different stress resistance test groups at 60°C. These results suggest a low overall abundance of *B*. *coagulans* and that multiple stress treatments (80°C for 10 min, pH 2.0, 0.1 mg/mL pepsin, and 0.3% pig bile salt) combined with a growth temperature of 55°C are required to enrich *B*. *coagulans* in the soil bacterial community. These results also indicate that *B*. *coagulans* is extremely resistant to various stresses (S2 Table).

The 12 *B*. *coagulans* strains, isolated from different sources, exhibited unique characteristics. The optimal temperature range of the 12 strains was between 45–55°C; that of 4 strains was 45°C, and that of 8 strains was 50°C. Moreover, the 12 *B*. *coagulans* strains showed no growth at pH 3.5, rather, 11 strains grew slowly at pH 4.5, and 7 strains grew optimally at pH 7.0; pH range 5.5–8.5 had the optimal growth condition. All were facultative anaerobic strains that produced lactic acid; six strains produced xylanase, and another six produced α-galactosidase (S3 Table). Although *B*. *coagulans* X26 grew well at 45–55°C, its growth rate was slightly faster at 50°C. Meanwhile, *B*. *coagulans* X26 grew considerably slower with a long lag period at 37°C and did not grow at 28°C (S1A Fig). The optimal pH for *B*. *coagulans* X26 growth was 7.0 (S1B Fig); however, the strain tolerated 0.1 mg/mL 7012 gastric acid or 0.3% pig bile salt at pH 2.0. *B*. *coagulans* X26 produced lactic acid at a rate of 0.3655 g/g and also produced xylanase and *α*-galactosidase.

The 12 strains also had different optimal carbon sources. Although all 12 strains utilized glucose, 4 strains preferred glucose, 2 preferred lactose, 2 preferred sucrose, 2 preferred maltose, and the final 2 strains preferred bran powder. The biomass of *B*. *coagulans* G4 was highest on the basal medium ([5.16 ± 0.24] × 10^8^ CFU/mL), followed by *B*. *coagulans* X26 ([5.15 ± 0.1] × 10^8^ CFU/mL). The yield of *B*. *coagulans* X26 was the highest on the optimal carbon source (maltose) ([5.28 ± 0.30] × 10^8^ CFU/mL).

*B*. *coagulans* X26 exhibited advantages over the other strains in enzyme production, acid production, and biomass (S3 and S4 Tables). Due to its enzyme-producing ability, *B*. *coagulans* X26 may aid in host digestion; its production of acid may also improve the content of short-chain fatty acids to regulate the intestinal tract, while its high level of biomass may reduce the cost of its application.

### Functional evaluation of *B*. *coagulans X26*

To further elucidate its probiotic effect, rats were fed *B*. *coagulans* X26, and their body weights were recorded weekly and compared to those in the oxytetracycline (a widely used antibiotic), *B*. *licheniformis*, and *E*. *faecium* treatment groups. During the feeding period, none of the rats had abnormal health condition. After feeding for 1 week, the body weights in the oxytetracycline, *B*. *coagulans*, and *B*. *licheniformis* groups were comparable to those in the control group, whereas those in the *E*. *faecium* group were significantly lower, which may be related to the gastric acid tolerance of *E*. *faecium*. However, after 2 weeks of feeding, the *B*. *coagulans* X26 group gained significantly more weight than the rats fed with other bacteria or the antibiotic, thereby indicating that *B*. *coagulans* X26 has a significant positive effect on rat growth (Table 1). As previously reported, *B*. *coagulans* has characteristics of *Bacillus* and *Lactobacillus* species [22] in that it is highly resistant to high temperatures, gastric acid, and bile salts, and produces lactic acid, which may explain its superiority in promoting rat growth.

**Table 1:** Body weight of rats supplemented with different *Bacillus* spp. or an antibiotic.

Test group	Original weight (g)	1-week feeding (g)	2-week feeding (g)	3-week feeding (g)
**Control**	175.6 ± 12.01	219.0 ± 7.42^a^	262.0 ± 11.51^b^	343.2 ± 12.49
**Antibiotic**	168.0 ± 7.21	216.0 ± 13.42^ab^	274.0 ± 23.02a^b^	338.83 ± 37.75
***B*. *coagulans***	174.0 ± 8.46	213.0 ± 5.70^ab^	292.0 ± 10.37^a^	360.7 ± 20.62
***B*. *licheniformis***	174.0 ± 6.52	205.4 ± 11.82^ab^	265.0 ± 19.04^b^	341.85 ± 32.04
***E*. *faecium***	174.6 ± 3.65	203.0 ± 7.58^b^	263.6 ± 19.17^b^	338.9 ± 34.85

In the same column, values with the same or no superscript letters indicate no significant difference (*p* ≥ 0.05); those with different lowercase superscript letters indicate a significant difference (*p* < 0.05).

Based on its superior performance in terms of biomass, acid production, enzyme production, and probiotic function, *B*. *coagulans* X26 was selected to optimize the fermentation process, which is expected to reduce production costs and facilitate its broad application.

### *Bacillus coagulans* X26 liquid fermentation medium and process optimization

#### Single-factor test results

According to the single-factor screening experiments, the top three production factors among carbon source, nitrogen source, and trace elements were selected as factors for PB design optimization. The preferred carbon sources and yields were: lactose (5.18 ± 0.39) × 10^8^ CFU/mL, glucose (5.15 ± 0.16) × 10^8^ CFU/mL, and maltose (5.28 ± 0.17) × 10^8^ CFU/mL; preferred nitrogen sources and yields were: corn steep liquor (3.62 ± 0.1) × 10^8^ CFU/mL, corn gluten powder (3.06 ± 0.07) × 10^8^ CFU/mL, and yeast extract (2.97 ± 0.13) × 10^8^ CFU/mL. The other factors and yields were: dipotassium hydrogen phosphate (5.85 ± 0.25) × 10^8^ CFU/mL, glutamic acid (5.5 ± 0.22) × 10^8^ CFU/mL, and manganese sulfate (4.1 ± 0.1) × 10^8^ CFU/mL.

#### PB design key factor screening

The PB experimental design and results are shown in Table 2. We used a 11-factor analysis table to perform variance and code equation analysis. The analysis of variance is presented in Table 3. The model F value was 9.32, which represents significance. Model terms with *p* < 0.05 were significant; factors C, D, G, H, J, and K were identified as significant model terms. Eq (1) shows that the response value caused by factors G, C, and D had the largest fluctuation range, and the prompt has the greatest impact on the response value. Factors G, C, and D were set as the key analysis factors.


R1=27.36–1.25B+1.8C+1.71D–1.11F+4.0G+1.6H+0.395I+1.7J–1.52K (1)

**Table 2:** Plackett-Burman (PB) design and results.

Run	A	B	C	D	E	F	G	H	I	J	K	Yield (×10^8^ CFU/mL)
	Blank	Glucose	Maltose	K_2_HPO_4_	Blank	Corn gluten meal	Yeast extract	Corn steep liquor	MnSO_4_	Lactose	Glutamate	
1	-1	-1	1	-1	1	1	-1	1	1	1	-1	28.75 ± 3.19
2	0	0	0	0	0	0	0	0	0	0	0	39.08 ± 2.70
3	1	-1	1	1	-1	1	1	1	-1	-1	-1	36.09 ± 2.04
4	1	1	-1	1	1	1	-1	-1	-1	1	-1	22.43 ± 7.07
5	1	1	1	-1	-1	-1	1	-1	1	1	-1	33.38 ± 6.22
6	-1	1	-1	1	1	-1	1	1	1	-1	-1	32.88 ± 0.94
7	1	-1	-1	-1	1	-1	1	1	-1	1	1	31.89 ± 2.97
8	-1	1	1	1	-1	-1	-1	1	-1	1	1	27.83 ± 5.20
9	1	-1	1	1	1	-1	-1	-1	1	-1	1	25.12 ± 2.76
10	1	1	-1	-1	-1	1	-1	1	1	-1	1	16.32 ± 0.28
11	-1	1	1	-1	1	1	1	-1	-1	-1	1	23.82 ± 3.27
12	-1	-1	-1	-1	-1	-1	-1	-1	-1	-1	-1	19.74 ± 2.87
13	-1	-1	-1	1	-1	1	1	-1	1	1	1	30.10 ± 4.79

**Table 3:** Plackett-Burman (PB) design analysis of variance table.

Source	Sum of squares	df	Mean square	F value	*p* value
**Model**	1182.54	9	131.39	9.32	< 0.0001
**B-B**	56.3	1	56.3	3.99	0.0555
**C-C**	117	1	117	8.3	0.0075
**D-D**	105.4	1	105.4	7.48	0.0107
**F-F**	44.4	1	44.4	3.15	0.0868
**G-G**	575.04	1	575.04	40.79	< 0.0001
**H-H**	91.84	1	91.84	6.51	0.0164
**I-I**	5.62	1	5.62	0.3984	0.533
**J-J**	104.24	1	104.24	7.39	0.0111
**K-K**	82.69	1	82.69	5.87	0.0222
**Curvature**	380.2	1	380.2	26.97	< 0.0001
**Residual**	394.72	28	14.1		
**Lack of fit**	1.63	2	0.8127	0.0538	0.9478
**Pure error**	393.09	26	15.12		
**Cor. total**	1957.45	38			
**R^2^**	0.9775				
**Adjusted R^2^**	0.876				

Model terms with *p* < 0.05 are significant. Cor. total: corrected total. df: degree of freedom.

#### Steepest slope experimental results

After six ramps, the yields were (39.08 ± 1.02) × 10^8^ CFU/mL, (40.12 ± 2.11) × 10^8^ CFU/mL, (40.04 ± 2.05) × 10^8^ CFU/mL, (39.38 ± 1.28) × 10^8^ CFU/mL, (39.03 ± 3.12) × 10^8^ CFU/mL, and (38.95 ± 2.78) × 10^8^ CFU/mL. The spore yield no longer increased after two climbing steps, indicating that the current test conditions were relatively optimal. Therefore, the second climbing value was used as the center point in the subsequent center combination design for further optimization (maltose: 16.5 g/L; yeast extract: 25.00 g/L; and K_2_HPO_4_: 3.5 g/L).

#### CCD design results

Based on the CCD (Table 4) with maltose (A), yeast extract (B), and K_2_HPO_4_ (C) as the independent variables and the number of viable bacteria in the fermentation broth (R) as the dependent variable, a quadratic regression model was established (Eq (2)), with a regression coefficient of R^2^ = 0.9960, representing a significant regression.


$$R=38.33+1.38A–4.33B–6.82C+0.39AB–0.41AC+3.75BC–0.45A^{2}–2.74B^{2}–0.44C^{2}$$ (2)

**Table 4:** Center combination design (CCD).

Run	Factor 1	Factor 2	Factor 3	Response (×10^8^ CFU/mL)
	A:A	B:B	C:C	R
**1**	0	0	0	39.03 ± 1.02
**2**	0	0	0	38.36 ± 1.24
**3**	1	-1	-1	51.27 ± 2.67
**4**	-1	-1	-1	47.57 ± 2.05
**5**	0	0	0	37.56 ± 1.11
**6**	0	0	-1.68179	49.00 ± 1.35
**7**	1	1	1	28.68 ± 1.40
**8**	0	0	0	38.29 ± 2.21
**9**	0	0	1.68179	26.05 ± 1.55
**10**	0	0	0	38.48 ± 1.12
**11**	0	1.68179	0	24.06 ± 1.85
**12**	0	-1.68179	0	38.00 ± 1.23
**13**	-1	1	1	25.06 ± 1.65
**14**	-1	-1	1	27.90 ± 1.80
**15**	0	0	0	38.10 ± 1.54
**16**	1	-1	1	28.70 ± 1.32
**17**	-1	1	-1	31.00 ± 1.35
**18**	1	1	-1	35.00 ± 1.22
**19**	-1.68179	0	0	35.50 ± 1.35
**20**	1.68179	0	0	39.50 ± 1.47

**Fig 1:**
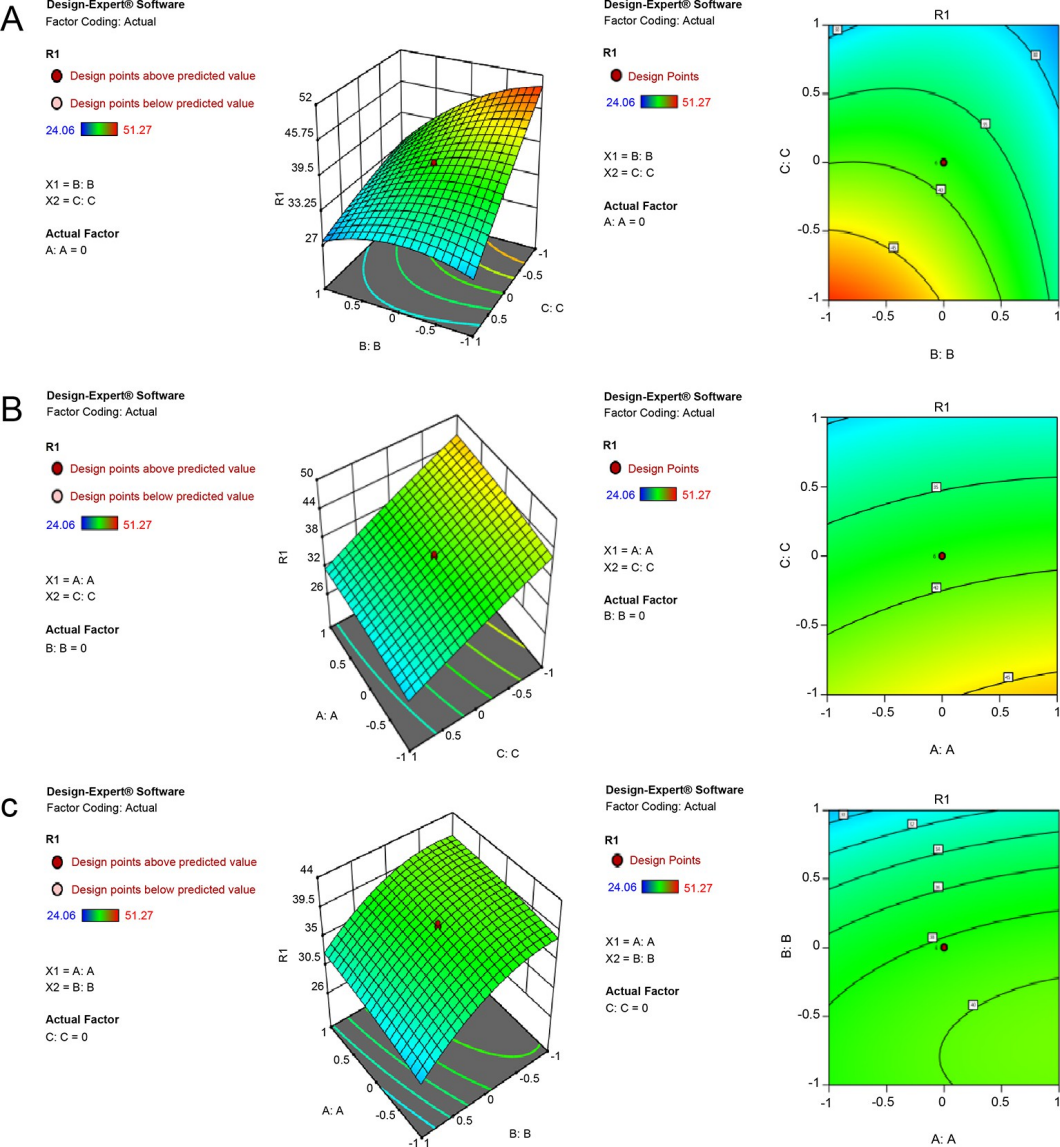
Contour plots and response surface plots. (A) Effect of the interaction of yeast extract and K_2_HPO_4_ on the number of viable bacteria when the amount of maltose is zero (16.5 g/L). (B) Effect of the interaction of maltose and K_2_HPO_4_ on the number of viable bacteria when the amount of yeast extract is zero (25 g/L). (C) Effect of the interaction of maltose and yeast extract on the number of viable bacteria when the amount of K_2_HPO_4_ is zero (3.5 g/L).

The response surface in Fig 1A was relatively steep, indicating that B and C had a significant effect on the number of viable bacteria, and its contour map was approximately elliptical, indicating that their interaction was also significant. Based on the contour and response surface maps, at a maltose concentration of 16.5 g/L, the number of viable bacteria first increased and then decreased with increasing yeast extract content and increased with decreasing K_2_HPO_4_ content.

Moreover, the response surface in Fig 1B was relatively steep, indicating that A and C had a significant effect on the number of viable bacteria; however, the contour map was not elliptical, thereby indicating that their interaction was not significant. The contour and response surface maps show that at a yeast extract content of 25 g/L, the number of viable bacteria increased with increasing maltose content and decreasing K_2_HPO_4_ content.

The response surface in Fig 1C was relatively steep, indicating that A and B had a significant effect on the number of viable bacteria, and its contour map was not elliptical, indicating that their interaction was not significant. The contour and response surface maps show that at a 3.5 g/L K_2_HPO_4_ content, the number of viable bacteria cells increased with increasing maltose content and decreasing yeast extract content.

The quadratic surfaces of A, B, and C had extreme values, and the openings of the response surfaces were all downward, indicating that the fermentation formula could be optimized through this response surface optimization test to obtain the highest effect of viable bacteria count.

We analyzed the variance in the regression model (Table 5) and found that (1) the regression model F value was 278.06 and *p* < 0.0001, representing extreme significance. (2) R^2^ = 0.9960 and R^2^_Adj_ = 0.9924 represent a strong correlation, good model fit, and small experimental error. (3) The lack of fit term (0.1308) had a *p* > 0.05, representing good model stability, a small proportion of abnormal error in actual fitting, and overall good model fit. (4) The CV value was 1.88%, representing a high confidence level for the model, which can be used to analyze the change in the response value.

**Table 5:** Analysis of variance (ANOVA) of the regression model.

Source	Sum of Squares	df	Mean Square	F value	*p* value	Significance
**Model**	1140.28	9	126.70	278.06	< 0.0001	**
**A-A**	26.01	1	26.01	57.08	< 0.0001	**
**B-B**	256.14	1	256.14	562.14	< 0.0001	**
**C-C**	634.63	1	634.63	1392.81	< 0.0001	**
**AB**	1.22	1	1.22	2.67	0.1333	
**AC**	1.34	1	1.34	2.95	0.1166	
**BC**	112.35	1	112.35	246.57	< 0.0001	**
**A^2^**	2.89	1	2.89	6.34	0.0305	*
**B^2^**	107.81	1	107.81	236.60	< 0.0001	**
**C^2^**	2.77	1	2.77	6.09	0.0332	*
**Residual**	4.56	10	0.4556			
**Lack of Fit**	3.40	5	0.6800	2.94	0.1308	
**Pure Error**	1.16	5	0.2313			
**Cor Total**	1144.84	19				
R^2^ = 0.9960		Adjusted R^2^ = 0.9924		Predicted R^2^ = 0.9750		CV = 1.88%

* *p* < 0.05

** *p* < 0.01.

For the *p*-value, the primary terms A, B, and C, the interaction term BC, and the quadratic term B^2^ had significant effects on the number of viable bacteria (*p* < 0.01); the effects of the quadratic terms A^2^ and C^2^ were also significant (*p* < 0.05). Moreover, the F value of each test factor reflected its degree of influence on the response value; with an increasing F value, the effect of the test factor on the response value increased. Given that F_A_ = 57.08, F_B_ = 562.14, and F_C_ = 1392.81, the order of influence for these three factors on the number of viable bacteria was K_2_HPO_4_ (C) > yeast extract (B) > maltose (A).

The above analysis indicated that the model-predicted value maintained a high degree of consistency with the actual value, indicating that the model was successfully established and statistically significant. The model was analyzed using Design Expert 11, and the optimal ratio was obtained as maltose 18.0 g/L, yeast extract 20.00 g/L, and K_2_HPO_4_ 3.0 g/L; the highest predicted yield was 5.1 × 10^9^ CFU/mL.

### Liquid fermentation verification and process optimization

The yield under the optimized culture conditions was verified by 500-mL shake flask fermentation. The number of viable bacteria measured after 36 h of culture was (5.12 ± 0.01) × 10^9^ CFU/mL, which was consistent with the prediction results of the model, confirming good model accuracy and validity. Compared with that in the basal medium, the number of viable bacteria in the optimized medium increased by 9.9 times. A 30-L experimental tank was used to verify the CCD-optimized medium, and the measured viable bacteria count was (5.11 ± 0.02) × 10^9^ CFU/mL, which was consistent with the 500-mL shake flask results and model prediction.

**Fig 2:**
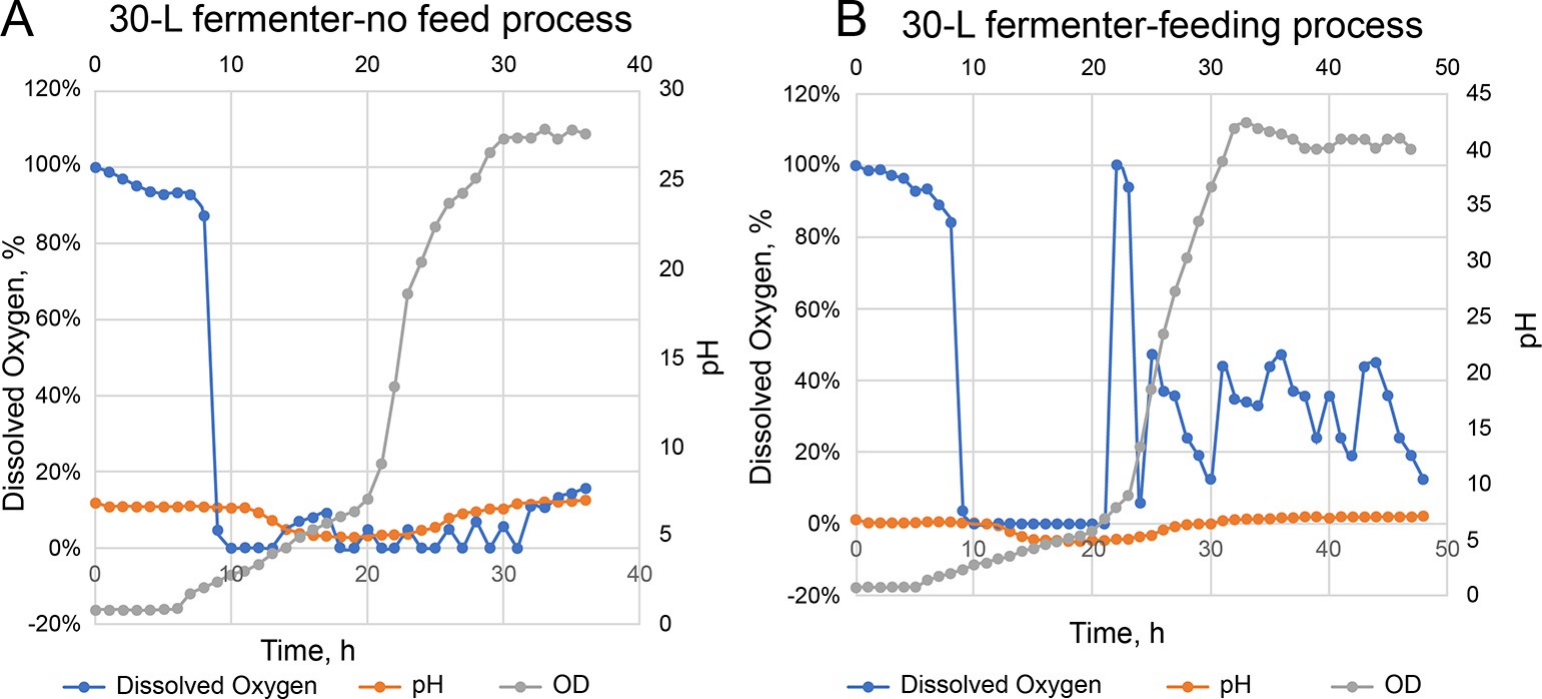
30-L fermentation process. (A) 30-L fermenter process without feeding. (B) Feed process.

In the early stage of fermentation, the cells grew rapidly, dissolved oxygen levels decreased rapidly, OD_600_ levels increased, and pH first decreased before it rebounded (Fig 2). Bacterial autolysis occurred in the later stage of fermentation, and the spore rate was relatively low. To prevent bacterial autolysis, we added glucose and yeast extract and controlled the dissolved oxygen level at 10–50% in the late logarithmic phase of bacterial growth before the stable phase, which increased the number of viable bacteria to (7.8 ± 0.2) × 10^9^ CFU/mL. The spore rate reached 96.54%, and the number of viable bacteria increased by 1.53-fold before the adjustment. According to the culture differences between the 10-T production tank and the 30-L fermenter, we adjusted the process conditions and guaranteed parameters and found that the number of viable bacteria reached (8.7 ± 0.1)×10^9^ CFU/mL with a 97.93% spore rate, which was 1.12-fold higher than that in the 30-L experimental tank.

The fermentation broth had a solid content of approximately 4% and was cooled to 25°C, concentrated approximately five times using a disc centrifuge, mixed with 15% starch and 5% protective agent as a carrier, and spray-dried while stirring to prepare a powdered *B*. *coagulans* X26; wherein the number of viable bacteria was (1.40 ± 0.1) × 10^11^ CFU/mL, and the spore rate was > 98%.

## Discussion

In this study, we screened *B*. *coagulans* X26 from soil under varying culture conditions and found that it showed a high level of resistance to multiple stressors, strong enzyme and acid production capacity, and high biomass yield. Meanwhile, *B*. *coagulans* X26 also improved the body weights of rats and the growth performance of laying hens [23], thereby highlighting its potential for applications in commercial animal husbandry.

Response surface design is a widely used optimization method. In fact, Chen et al. applied this method to increase the yield of *B*. *subtilis* WHK from (2.7 ± 0.75)×10^9^ to (1.52 ± 0.06)×10^10^ CFU/mL [24]. Additionally, Sun et al. adopted the response surface design to optimize the fermentation conditions of *Pichia pastoris* GS115 to increase the production of xylanase. After optimization, the production increased by 2.1-fold [25]. In the present study, we utilized response surface design to determine that the main nutritional factors affecting the X26 yield were yeast extract, maltose, and K_2_HPO_4_. After optimization of the medium, the yield increased by 9.9-fold. After process adjustment, the yield further increased by 1.53-fold. These results indicated that the yield improvement through process adjustment was lower than that of the previous medium optimization. However, adjustment of the post-fermentation process had a positive effect on improving the spore rate.

In this study, at a fermentation temperature of 45°C, the viable count in the 500 mL shake flask was (5.12 ± 0.01) × 10^9^ CFU/mL, while that in the 30-L fermenter was (7.8 ± 0.2) × 10^9^ CFU/mL, the number of spores was 7.53 × 10^9^, and the spore rate was 96.54%. Similarly, within the 10-T fermenter, the number of viable bacteria was (8.7 ± 0.1) × 10^9^ CFU/mL, the number of spores was 8.52 × 10^9^ CFU/mL, and the spore rate was 97.93%. Meanwhile, Dong et al. fermented *B*. *coagulans* BLCC1-0517 at 42°C for 48 h using an optimized medium, and achieved a spore density of 3.38 × 10^8^ CFU/mL [26]. Li et al. cultured *B*. *coagulans* CMCC337209 in optimized conditions at 45°C for 96 h to obtain a maximum viable count of 1.15 × 10^9^ CFU/mL and a spore count of 1.09 × 10^9^ CFU/mL [14]. Sun et al. used high-density fermentation to culture *B*. *coagulans* CGMCC NO.7431 and generated a viable bacteria number of 3.095 × 10^9^ CFU/mL at 45°C for 16 h, although spore yield was not reported [15]. Ding used high-density fermentation to optimize the yield of *B*. *coagulans* CNJD and generate a viable bacteria number of 3.2 × 10^12^ CFU/mL; however, only 4.3 × 10^9^ CFU/mL spores were obtained, corresponding to a spore rate < 1% [16]. Qiu et al. evaluated the fermentation characteristics of *B*. *coagulans* T-8 cultured at 47°C for 28 h and obtained a viable count of 4.8 × 10^9^ CFU/mL and spore count of 4.57 × 10^9^ CFU/mL [17]. Hence, at a fermentation temperature of 45°C, the spore amounts obtained in the current study in the 30-L (7.53 × 10^9^ CFU/mL) and 10-T fermenter tanks (8.52 × 10^9^ CFU/mL) were the highest reported values for *B*. *coagulans* fermentation at this temperature (S5 Table).

In industrial production, the efficient recovery of live bacteria at low costs is the main challenge when developing high-content live bacteria preparations; two processes are commonly utilized. The first is freeze-vacuum drying which can be used for live bacteria or spores; however, its costs are high, thereby limiting its application in the animal husbandry industry. The second is the spray drying process, which is simple to perform and cost-effective. However, the high temperatures during the drying process kill cells and result in a low bacterial yield. To address this problem, we optimized the fermentation process to achieve a spore rate > 95% and considered the temperature tolerance of the spores to avoid extreme cell losses during the drying process. The screening of appropriate carriers and protective agents can further improve the dry yield of viable bacteria to 90%. Indeed, we prepared (1.40 ± 0.1) × 10^11^ CFU/mL *B*. *coagulans* powder, thus, confirming that *B*. *coagulans* X26 not only has probiotic properties, but also an appropriate yield capacity for industrial fermentation.

## Conclusions

Our study describes an optimized industrial development protocol for *B*. *coagulans* X26, including strain screening, performance evaluation, effect evaluation, and 10-T fermentation. Herein, we provided an empirical basis for the subsequent isolation of *B*. *coagulans* strains from the environment and optimization of culture conditions. Simultaneously, we have highlighted the importance of strain type as a factor that influences the fermentation temperature and biomass of *B*. *coagulans*. However, based on the limited number of *B*. *coagulans* strains and fermentation conditions, this study may also have certain limitations. Further studies are warranted to assess the relationship between growth temperature and fermentation yield.

## Supporting information

S1 Table Soil sample collection records. (DOCX)

S2 Table Natural screening results. (DOCX)

S3 Table Characteristics of different *B*. *coagulans* strains. (DOCX)

S4 Table Comparison of *B*. *coagulans* biomass. (DOCX)

S5 Table Fermentation levels of *B*. *coagulans* at different temperatures. (DOCX)

S1 Fig *B*. *coagulans* X26 growth curve. (TIF)

## References

[1] Zhou Y, Zeng Z, Xu Y, Ying J, Wang B, Majeed M, et al. Application of Bacillus coagulans in animal husbandry and its underlying mechanisms. Animals (Basel). 2020;10: 454. doi: 10.3390/ani10030454 32182789 PMC7143728

[2] Mu Y, Cong Y. Bacillus coagulans and its applications in medicine. Benef Microbes. 2019;10: 679–688. doi: 10.3920/BM2019.0016 31203635

[3] Konuray G, Erginkaya Z. Potential use of Bacillus coagulans in the food industry. Foods. 2018;7: 92. doi: 10.3390/foods7060092 29899254 PMC6025323

[4] Xinlei L. High cell and spore density culture of *L. sporogenes* (*Bacillus coagulans* TQ33) [D]. Tianjin University of Science and Technology. 2002. Available from: https://kns.cnki.net/KCMS/detail/detail.aspx?dbname=CMFD9904&filename=2003062174.nh.

[5] Wei Q, Xinlei L, Jianling W, Ying C, Fuping L, Lianxiang D. High-density cultivation of Bacillus coagulans TQ33 with hollow fiber filtration. Food Ferment Ind. 2003;29: 15–18.

[6] Dongliang C, Jiangming T, Yunshan W, Liping Z. The elementary study of industrial culture medium of *Bacillus coagulans* fermentation. Food Ferment Ind. 2007;12: 73–75.

[7] Qiuhong C, Mei S, Qun K, Dalin S, Huai L, Linghong H, et al. Influence of cultivation condition on sporulation of *Bacillus coagulans*. Biotechnology. 2009;19: 77–81. doi: 10.16519/j.cnki.1004-311x.2009.01.012

[8] Shufeng G, Lihua K, Yinghua Z, Xinxu H, Shenglian W, Dong M, et al. The influence on fermentation level of *Bacillus coagulans* by adopting segmented fed-batch fermentation technology. China Anim Husbandry Vet Med. 2013;40: 80–84.

[9] Nan Z. Study of the high cell density fermentation of *Bacillus coagulans* [D]. Tianjin University of Science and Technology. 2016. Available from: https://kns.cnki.net/kcms2/article/abstract?v=3uoqIhG8C475KOm_zrgu4lQARvep2SAkueNJRSNVX-zc5TVHKmDNktzPjs4xumcSQdWt9Y1ncAhM7N8iUhLOOM11dPs9uPBd&uniplatform=NZKPT&src=copy.

[10] Pandey KR, Vakil BV. Development of bioprocess for high density cultivation yield of the probiotic *Bacillus coagulans* and its spores. J BioSci Biotechnol. 2016;5: 173–181.

[11] Lina Z. Screening of probiotics *Bacillus coagulans* and optimization of its high density cultivation conditions [D]. Henan University of Science and Technolog. 2017. Available from: https://kns.cnki.net/KCMS/detail/detail.aspx?dbname=CMFD201801&filename=1017829671.nh.

[12] Biao S, Weijing C, Yingqing Z, Jianjun L, Yinfang T, Fuming Z. Optimization of the spore-forming and high density culture conditions for *Bacillus coagulans*. J Hunan Agric Univ (Nat Sci). 2021;47: 171–179. doi: 10.13331/j.cnki.jhau.2021.02.008

[13] Yin L, Chen MX, Zheng TH, Liu XM, Zhu F, Huang RQ. Improving probiotic spore yield using rice straw hydrolysate. Lett Appl Microbiol. 2021;72: 149–156. doi: 10.1111/lam.13387 32939775

[14] Kaixiao L, Miao L, Shuaibiao L, Wanqiu Y, Hongfei M, Zhenzhen Z, et al. Optimized culture control for producing spores of *Bacillus coagulans*; 83–87; 2019. doi: 10.26914/c.cnkihy.2019.07106 [in Chinese].

[15] Lina S, Xun J, Quanxing Z, Jinsong Z, Dongmei L. High cell density culture of *Bacillus* 13002. Food Ind Technol. 2017;38: 114–120. doi: 10.13386/j.issn1002-0306.2017.21.024

[16] Honghao D. Screening of *Bacillus coagulans* and high-density fermentation [D]. Hubei University of Technology. 2021. Available from: https://kns.cnki.net/kcms2/article/abstract?v=3uoqIhG8C475KOm_zrgu4lQARvep2SAkueNJRSNVX-zc5TVHKmDNkqT7prPMUNk2tOj-RfOEu5WwKkK1RYb5QFX2VMfEToRx&uniplatform=NZKPT&src=copy.

[17] Yingying Q, Yanzhi L, Danling H, Nanxi L, Yi Y, Chengqun C. Screening and characterization of probiotics for fodder fermentation. Journal of Guangdong Industry Polytechnic. 2022;21: 1–7. doi: 10.13285/j.cnki.gdqgxb.2022.0001

[18] Chauhan S, Choudhury B, Singh SN, Ghosh P. Application of xylanase enzyme of *Bacillus coagulans* as a prebleaching agent on non-woody pulps. Process Biochem. 2006;41: 226–231. doi: 10.1016/j.procbio.2005.06.003

[19] Zhao R, Zhao R, Tu Y, Zhang X, Deng L, Chen X. A novel α-galactosidase from the thermophilic probiotic Bacillus coagulans with remarkable protease-resistance and high hydrolytic activity. PLoS ONE. 2018;13: e0197067. doi: 10.1371/journal.pone.0197067 29738566 PMC5940202

[20] Mazkour S, Shekarforoush SS, Basiri S. The effects of supplementation of Bacillus subtilis and Bacillus coagulans spores on the intestinal microflora and growth performance in rat. Iran J Microbiol. 2019;11: 260–266. 31523411 PMC6711872

[21] Gharib AA, El-Hamid MIA, El-Aziz NKA, Yonan EY, Allam MO. Bacillus cereus: Pathogenicity, viability and adaptation. Adv Anim Vet Sci. 2020;8: 34–40. doi: 10.17582/journal.aavs/2020/8.s1.34.40

[22] Drago L, De Vecchi E. Should Lactobacillus sporogenes and Bacillus coagulans have a future? J Chemother. 2009;21: 371–377. doi: 10.1179/joc.2009.21.4.371 19622453

[23] Xu L, Zhou Y, Zhan Z, Zhang W, Fu D, Zhao R, et al. Research Note: Effects of Bacillus coagulans X26 on the production performance, intestinal structure, short-chain fatty acids and flora composition of laying hens during the peak laying period. Poult Sci. 2022;101: 101835. doi: 10.1016/j.psj.2022.101835 35398755 PMC9006320

[24] Chen ZM, Li Q, Liu HM, Yu N, Xie TJ, Yang MY, et al. Greater enhancement of Bacillus subtilis spore yields in submerged cultures by optimization of medium composition through statistical experimental designs. Appl Microbiol Biotechnol. 2010;85: 1353–1360. doi: 10.1007/s00253-009-2162-x 19697022

[25] Sun T, Yan P, Zhan N, Zhang L, Chen Z, Zhang A, et al. The optimization of fermentation conditions for pichia pastoris GS115 producing recombinant xylanase. Eng Life Sci. 2020;20: 216–228. doi: 10.1002/elsc.201900116 32874185 PMC7447871

[26] Peipei D, Xiangyan W, Yuanxiang L, Guoqin X, Haiyan X, Wei G, et al. Optimization of fermentation medium of *Bacillus coagulans*. China Brew. 2018;37: 28–32. doi: 10.11882/j.issn.0254-5071.2018.04.006

