# Supplementary material for: Isolation, functional evaluation, and fermentation process optimization of probiotic Bacillus coagulans
Source: PLoS One. 2023 Nov 3;18(11):e0286944. doi: 10.1371/journal.pone.0286944 (PMC10624278; doi:10.1371/journal.pone.0286944)
Supplement: S1 Table — (DOCX) [file pone.0286944.s001.docx]

**S1 Table Soil sample collection records.**

| **Number** | **Area** | **Location** |
| --- | --- | --- |
| **1** | Berlin Cooperative in Mayingbao Village, Shuocheng District, Shuozhou, Shanxi | Garlic yellow ground |
| **2** | Berlin Cooperative in Mayingbao Village, Shuocheng District, Shuozhou, Shanxi | Peach field |
| **3** | Berlin Cooperative in Mayingbao Village, Xiatuan Township, Shuocheng District, Shuozhou, Shanxi | Landscape tree |
| **4** | Greenhouse in Yehujian Village, Shuocheng District, Shuozhou, Shanxi | Cucumber field |
| **5** | Greenhouse in Yehujian Village, Shuocheng District, Shuozhou, Shanxi | Tomato field |
| **6** | Greenhouse in Yehujian Village, Shuocheng District, Shuozhou, Shanxi | Grape field |
| **7** | Greenhouse in Yehujian Village, Shuocheng District, Shuozhou, Shanxi | Mushroom field |
| **8** | Jiangxi Wuyuan | Vegetable plot |
| **9** | Heshan, Guangdong | Vegetable plot |
| **10** | Ningxia Yinchuan | Vegetable plot |
| **11** | Tianmen Village, Wuzu Town, Huangmei County, Huanggang City, Hubei Province | Negetable plot |
| **12** | Shihezi, Xinjiang | Vegetable plot |
| **13** | Chengdu, Sichuan | Vegetable plot |
| **14** | Tomato Field in Jiujie Town, Xinzhou, Wuhan, Hubei | Tomato field |
