# Supplementary material for: Isolation, functional evaluation, and fermentation process optimization of probiotic Bacillus coagulans
Source: PLoS One. 2023 Nov 3;18(11):e0286944. doi: 10.1371/journal.pone.0286944 (PMC10624278; doi:10.1371/journal.pone.0286944)
Supplement: S2 Table — (DOCX) [file pone.0286944.s002.docx]

**S2 Table** **Natural screening results.**

| **Resistant conditions** | **Culture medium** | **Strains** | | |
| --- | --- | --- | --- | --- |
|  |  | **Culture temperature**  **37 ℃** | **Culture temperature**  **55 ℃** | **Culture temperature**  **60 ℃** |
| **pH 7.2 YPD** | NA | *B. cereus* | *B. stutzeri, B. licheniformis, B. paralicheniformis, B. soy fermentum, B. subtilis, B. tequila* | *B. stutzeri* |
|  | MRS5.5 | *B. siamese, B.amyloliquefaciens, B. veles* | *B. stutzeri* | *B. stutzeri* |
| **0.1 mg/mL pepsin, pH 2.0 YPD** | NA | *B. cereus, Staphylococcus scalp* | *B. stutzeri* | *B. stutzeri* |
|  | MRS5.5 | *B. siamese, B. amyloliquefaciens, B. veles* | *B. stutzeri* | *B. stutzeri* |
| **0.3% bile salts, 0.1 mg/mL pepsin, pH 2.0 YPD** | NA | *B. subtilis, B. tequila, B. cereus, B. licheniformis, B. paralicheniformis, B. sonora desert* | *B. stutzeri*, *B. coagulans* X26 | *B. stutzeri* |
|  | MRS5.5 | *B. siamese, B. amyloliquefaciens, B. veles* | *B. stutzeri*, *B. coagulans* X60 | *B. stutzeri* |
