# Supplementary material for: Isolation, functional evaluation, and fermentation process optimization of probiotic Bacillus coagulans
Source: PLoS One. 2023 Nov 3;18(11):e0286944. doi: 10.1371/journal.pone.0286944 (PMC10624278; doi:10.1371/journal.pone.0286944)
Supplement: S3 Table — (DOCX) [file pone.0286944.s003.docx]

**S3 Table Characteristics of different *B. coagulans* strains.**

| **Strain** | **Source** | **Growth temperature** | **pH** | **Lactic acid conversion rate (g/g)** | **Xylanase** | **α-galactosidase** |
| --- | --- | --- | --- | --- | --- | --- |
| **ATCC 7050** | China center for type culture collection | Grows slowly at 28 ℃; can grow at 37 ℃; grows well at 45–50 ℃; optimal growth at 45 ℃, 45 ℃ > 50 ℃ > 55 ℃. | No growth at pH 3.5; slow growth at 4.5; good growth at 5.5–8.5; optimal growth at pH 8.5. | 0.1025 | + | + |
| **GDMCC 1.645** | Guangdong Microbial Culture Collection center | Grows slowly at 28 ℃; can grow at 37 ℃; grows well at 45–55 ℃, 50 ℃ > 45 ℃ > 55 ℃. | No growth at pH 3.5; slow growth at 4.5; good growth at 5.5–8.5; optimal growth at pH 7.0. | 0.3643 | – | – |
| **CGMCC**  **1.10823** | China General Microbiological Culture Collection Center | Slow growth at 28 ℃; good growth at 37 ℃; good growth at 45–55 ℃, 50 ℃ > 55 ℃ > 45 ℃. | No growth at pH 3.5; slow growth at 4.5; good growth at 5.5–7.5; slow growth at 8.5; optimal growth at pH 7.0. | 0.2032 | – | – |
| **G1** | Wuhan SunHY Biology Co.Ltd. | Slow growth at 28 ℃; good growth at 37 ℃; good growth at 50–55 ℃, 50 ℃ > 55 ℃ > 45 ℃. | No growth at pH 3.5; growth at 4.5 and 8.5; good growth at 5.5–7.5; optimal growth at pH 7.0. | 0.1325 | + | – |
| **G2** |  | Grows slowly at 28 ℃; can grow at 37 ℃; grows well at 50–55 ℃, 50 ℃ > 55 ℃ > 45 ℃. | No growth at pH 3.5; slow growth at 4.5; good growth at 5.5–7.5; low growth at 8.5; optimal growth at pH 7.0. | 0.1019 | – | + |
| **G3** | Sunhy Technology (Hubei) Co.，Ltd. | Grows slowly at 28 ℃; can grow at 37 ℃; grows well at 45–55 ℃, 45 ℃ > 50 ℃ > 55 ℃. | No growth at pH 3.5; lags in growth at 4.5; good growth at 5.5–8.5; optimal growth at pH 8.5. | 0.3608 | + | + |
| **G4** |  | Grows slowly at 28 ℃; can grow at 37 ℃; grows well at 45–55 ℃, 45 ℃ ≈ 50 ℃ > 55 ℃. | No growth at pH 3.5; slow growth at 4.5; good growth at 5.5–8.5; optimal growth at pH 8.5. | 0.2696 | + | – |
| **BNCC 188060** | [BeNa Culture Collection](https://www.bncc.org.cn/) | Grows slowly at 28 ℃; can grow at 37 ℃; grows well at 45–55 ℃, 50 ℃ > 45 ℃ ≈ 55 ℃. | Relatively no growth at pH 3.5–4.5; lags in growth at pH 5.5; good growth at pH 7.0–9.0; optimal growth at pH 8.5. | 0.1182 | + | + |
| **CICC21736** | China center of industrial culture collection | Slow growth at 28 ℃; grows well at 37 ℃, and at 50–55 ℃, 50 ℃ > 55 ℃ > 45 ℃. | No growth at pH 3.5; slow growth at 4.5; good growth at 5.5–8.5; optimal growth at pH 7.0. | 0.0917 | – | – |
| **ACCC 10229** | Agricultural culture collection of china | Grows slowly at 28 ℃; can grow at 37 ℃; grows well at 45–55 ℃, 45 ℃ ≈ 50 ℃ > 55 ℃. | No growth at pH 3.5; lags in growth at 4.5; good growth at 5.5–8.5; optimal growth at pH 8.5. | 0.1606 | – | – |
| **X26** | The screening results of this study | Grows slow at 28 ℃; grows well at 37 ℃, and at 45–55 ℃, 50 ℃ ≈ 45 ℃ ≈ 55 ℃. | No growth at pH 3.5; slow growth at 4.5; good growth at 5.5–8.5; optimal growth at pH 7.0 | 0.3655 | + | + |
| **X60** | The screening results of this study | Grows slow at 28 ℃; grows well at 37 ℃, and at 50–55 ℃, 50 ℃ > 55 ℃ > 45 ℃. | No growth at pH 3.5; slow growth at 4.5; good growth at 5.5–8.5; optimal growth at pH 6.5 | 0.1642 | – | + |

Note:“+” indicates that xylanase or α-galactosidase can be produced; “–” indicates that no xylanase or α-galactosidase can be produced.
