# Supplementary material for: Isolation, functional evaluation, and fermentation process optimization of probiotic Bacillus coagulans
Source: PLoS One. 2023 Nov 3;18(11):e0286944. doi: 10.1371/journal.pone.0286944 (PMC10624278; doi:10.1371/journal.pone.0286944)
Supplement: S4 Table — (DOCX) [file pone.0286944.s004.docx]

**S4 Table Comparison of *B. coagulans* biomass estimates.**

| **Strain** | **Glucose** | **Sucrose** | **Maltose** | **Lactose** | **Corn starch** | **Bran flour** | **Maltodextrin** | **Glucose** | **Sucrose** | **Maltose** | **Lactose** | **Corn starch** | **Bran flour** | **Maltodextrin** |
| --- | --- | --- | --- | --- | --- | --- | --- | --- | --- | --- | --- | --- | --- | --- |
|  | **Viable count (×10^8^ CFU/mL)** | | | | | | | **Spore rate** | | | | | | |
| **ATCC 7050** | 0.28 ± 0.01 | 4.1 ± 0.21 | 0.78 ± 0.01 | 0.55 ± 0.02 | 0.63 ± 0.01 | 0.03 ± 0.01 | 0.08 ± 0.01 | – | 5.37% | – | – | – | – | – |
| **GDMCC 1.645** | 0.6 ± 0.02 | 3.9 ± 0.03 | 3.1 ± 0.05 | 3.2 ± 0.19 | 4.2 ± 0.14 | 1.2 ± 0.02 | 0.72 ± 0.02 | 25.00% | 1.28% | – | 3.44% | 0.24% | – | 5.56% |
| **CGMCC1.10823** | 2.8 ± 0.06 | 3.4 ± 0.11 | 1.51 ± 0.02 | 3.6 ± 0.16 | 3.0 ± 0.13 | – | 0.16 ± 0.06 | 0.36% | 73.53% | – | – | – | – | 11.50% |
| **G1** | 4.7 ± 0.1 | 4.3 ± 0.12 | 2.2 ± 0.1 | 1.41 ± 0.01 | 2.86 ± 0.09 | 1.1 ± 0.01 | 0.22 ± 0.01 | 1.55% | 65.0% | – | – | – | – | 14.65% |
| **G2** | 1.25 ± 0.02 | 1.35 ± 0.02 | 1.2 ± 0.02 | 1.56 ± 0.11 | 2.42 ± 0.15 | 2.5 ± 0.03 | 1.47 ± 0.02 | – | – | 90.00% | 43.59% | 93.31% | 60.00% | 95.92 |
| **G3** | 5.1 ± 0.14 | 3.3 ± 0.15 | 2.23 ± 0.12 | 1.61 ± 0.04 | 2.27 ± 0.06 | 0.1 ± 0.01 | 0.82 ± 0.01 | 3.51% | 55.0% | 11.25% | – | – | – | 15.35% |
| **G4** | 5.16 ± 0.24 | 4.8 ± 0.34 | 4.6 ± 0.26 | 4.9 ± 0.27 | 5.1 ± 0.35 | 3.13 ± 0.23 | 2.8 ± 0.24 | – | 25.52 | – | – | 26.36% | – | 7.43 |
| **BNCC 188060** | 4.2 ± 0.11 | 2.35 ± 0.12 | 1.89 ± 0.08 | 3.89 ± 0.15 | 1.23 ± 0.09 | 0.15 ± 0.08 | 1.12 ± 0.07 | 11.24% | 18.2% | 4.27% | 10.25% | – | – | 2.37% |
| **CICC21736** | 2.2 ± 0.02 | 3.31 ± 0.14 | 4.81 ± 0.31 | 2.85 ± 0.03 | 1.28 ± 0.02 | 0.19 ± 0.02 | 1.78 ± 0.02 | 9.24% | – | – | 8.25% | – | – | – |
| **ACCC 10229** | 2.5 ± 0.12 | 3.56 ± 0.17 | 3.85 ± 0.12 | 3.88 ± 0.12 | 1.67 ± 0.04 | 0.22 ± 0.01 | 1.55 ± 0.12 | 10.55% | 12.70 | 2.56% | 10.2% | – | – | – |
| **X26** | 5.15 ± 0.16 | 2.12 ± 0.06 | 5.28 ± 0.17 | 5.18 ± 0.39 | 2.5 ± 0.11 | 2.14 ± 0.03 | 1.41 ± 0.04 | 23.7% | – | 47.4% | 46.0% | – | – | – |
| **X60** | 3.12 ± 0.2 | 1.56 ± 0.22 | 2.16 ± 0.17 | 4.5 ± 0.25 | 2.22 ± 0.20 | 3.485 ± 0.20 | 1.68 ± 0.09 | 2.35% | 1.85% | 11.04% | 1.55% | – | 1.05% | – |

Note: “–” indicates that the number of viable bacteria is < 0.01 × 10^8^ CFU/mL or the spore rate is < 0.01%.
