# Supplementary material for: Isolation, functional evaluation, and fermentation process optimization of probiotic Bacillus coagulans
Source: PLoS One. 2023 Nov 3;18(11):e0286944. doi: 10.1371/journal.pone.0286944 (PMC10624278; doi:10.1371/journal.pone.0286944)
Supplement: S5 Table — (DOCX) [file pone.0286944.s005.docx]

**S5 Table Fermentation levels of *B. coagulans* at different temperatures.**

| **S/N** | **Strain** | **Source** | **Reactor volume** | **Temperature and time** | **Growth at 45–55 ℃** | **Total bacterial count (CFU/mL)** | **Spore count (CFU/mL)** | **Spore rate (%)** | **Reference** |
| --- | --- | --- | --- | --- | --- | --- | --- | --- | --- |
| **1** | TQ33 | / | 5 L | 40 ℃, 26–48 h | – | 4.5 × 10^9^ | 1.2×10^9^ | 26.67 | [1] |
|  | TQ33 | / | 5 L | 40 ℃, 26–48 h | – | 1.5 × 10^10^ | 0.9×10^10^ | 60 |  |
| **2** | TQ33 | Skim milk powder | 5 L | 40 ℃, 48 h | – | 1.6 × 10^10^ | 1.2×10^10^ | 75 | [2] |
| **3** | TW-05 | / | 500 mL | 40 ℃, 36 h | – | 9.3 × 10^9^ | – | – | [3] |
| **4** | / | / | 15 L | 40 ℃, 20 h | The optimum temperature is 40 °C; the number of viable bacteria and spores decrease at ≥ 45 °C. | 6.0 × 10^9^ | 5.8 × 10^9^ | 96.7 | [4] |
| **5** | / | Hunan Institute of Microbiology | 30 L | 35 ℃, 42 h | – | 8.3 × 10^9^ | 7.11 × 10^9^ | 85.63 | [5] |
| **6** | / | / | 6.6 L | 37 ℃, 44 h | – | 3.8 × 10^11^ | 2.511 × 10^11^ | 81 | [6] |
| **7** | BC304 | Tianjin University of Science and Technology. | 250 mL | 37 ℃, 48 h | – | 9.5 × 10^9^ | 7.92 × 10^9^ | 83.37 | [7] |
| **8** | BC303 | Piglet intestines or feces. | – | 37 ℃, 20 h | The optimum temperature is 37 °C; biomass decreases at ≥ 40 °C. | 2.13 × 10^10^ | 8.4 × 10^8^ | 3.9 | [8] |
| **9** | CGMCC 1.2009 | / | 5 L | 30 ℃, 48 h | – | 6.17 × 10^11^ | 5.74 × 10^11^ | 93 | [9] |
| **10** | QR–N323 | Zhejiang Qirun Biotechnology Co., Ltd. | 500 L | 40 ℃, 36 h | The optimum temperature is 40 ℃; spore quantity decreased at 45 ℃. | 3.01 × 10^10^ | 2.98 × 10^10^ | 99.0 | [10] |
| **11** | X26 | Soil | 500 mL | 45 ℃, 24 – 48 h | The optimum temperature is 50 ℃; it can grow well at 45–55 ℃. | (5.12 ± 0.01) × 10^9^ | 2.61 × 10^9^ | 50.98 | This study |
|  |  |  | 30 L |  |  | (7.8 ± 0.2) × 10^9^ | 7.53 × 10^9^ | 96.54 |  |
|  |  |  | 10 T |  |  | (8.7 ± 0.1) × 10^9^ | 8.52 × 10^9^ | 97.89 |  |
| **12** | CGMCC  NO.7431 | Bean paste | 4 L | 45 ℃, 16 h | The optimum temperature is 45 °C; it can grow well at 50 °C. | 3.095 × 10^9^ | – | – | [11] |
| **13** | BLCC1-  0517 | Shandong Baolai Lilai Biological Engineering Co., Ltd. | / | 42 ℃, 48 h | – | – | 3.38 × 10^8^ | – | [12] |
| **14** | CMCC 337209 | China Culture Collection Center | 100 mL | 45 ℃, 96 h | The optimum temperature is 45 °C; it can grow well as 50 °C. | 1. 15 × 10^9^ | 1. 09 × 10^9^ | 94.78 | [13] |
| **15** | CNJD | Pickled water | 250 mL | 45–55 ℃, 16 h | The optimum temperature is 55 ℃; it can grow well at 45–55 ℃. | 7.86 × 10^10^ | – | – | [14] |
|  | CNJD | Pickled water | 30 L | 45 ℃, 36 h |  | 3.2 × 10^12^ | 4.3 × 10^9^ | 0.13 |  |
| **16** | T-8 | Feed | 500 mL | 47 ℃, 28 h | The optimum temperature is 47 ℃; it can grow well at 45–49 ℃. | 4.8 × 10^9^ | 4.57 × 10^9^ | 95.2 | [15] |

Note:“ –” indicates that the subject was not mentioned in the reference.

**REFERENCES**

1. Xinlei L. High cell and spore density culture of *L. sporogenes* (*Bacillus coagulans* TQ33) [D]. Tianjin University of Science and Technology. 2002. Available from: [https://kns.cnki.net/KCMS/detail/detail.aspx?dbname=CMFD9904&filename=2003062174.nh](https://ersp.lib.whu.edu.cn/s/net/cnki/kns/G.https/KCMS/detail/detail.aspx?dbname=CMFD9904&filename=2003062174.nh).
2. Wei Q, Xinlei L, Jianling W, Ying C, Fuping L, Lianxiang D. High-density cultivation of Bacillus coagulans TQ33 with hollow fiber filtration. Food Ferment Ind. 2003;29: 15–18..

3. Dongliang C, Jiangming T, Yunshan W, Liping Z. The elementary study of industrial culture medium of *Bacillus coagulans* fermentation. Food Ferment Ind. 2007;12: 73–75.

4. Qiuhong C, Mei S, Qun K, Dalin S, Huai L, Linghong H, et al. Influence of cultivation condition on sporulation of *Bacillus coagulans*. Biotechnology. 2009;19: 77–81. doi: [10.16519/j.cnki.1004-311x.2009.01.012](https://doi.org/10.16519/j.cnki.1004-311x.2009.01.012).

5. Shufeng G, Lihua K, Yinghua Z, Xinxu H, Shenglian W, Dong M, et al. The influence on fermentation level of *Bacillus coagulans* by adopting segmented fed-batch fermentation technology. China Anim Husbandry Vet Med. 2013;40: 80–84.

6. Pandey KR, Vakil BV. Development of bioprocess for high density cultivation yield of the probiotic *Bacillus coagulans* and its spores. J BioSci Biotechnol. 2016;5: 173–181.

7. Nan Z. Study of the high cell density fermentation of *Bacillus coagulans* [D]. Tianjin University of Science and Technology; 2016. doi: [10.27359/d.cnki.gtqgu.2016.000129](https://doi.org/10.27359/d.cnki.gtqgu.2016.000129). Available from: https://kns.cnki.net/kcms2/article/abstract?v=3uoqIhG8C475KOm_zrgu4lQARvep2SAkueNJRSNVX-zc5TVHKmDNktzPjs4xumcSQdWt9Y1ncAhM7N8iUhLOOM11dPs9uPBd&uniplatform=NZKPT&src=copy.

8. Lina Z. Screening of probiotics *Bacillus coagulans* and optimization of its high density cultivation conditions [D]. Henan University of Science and Technolog. 2017. Available from: [https://kns.cnki.net/KCMS/detail/detail.aspx?dbname=CMFD201801&filename=1017829671.nh](https://ersp.lib.whu.edu.cn/s/net/cnki/kns/G.https/KCMS/detail/detail.aspx?dbname=CMFD201801&filename=1017829671.nh).

9. [Yin L](https://www.citexs.com/Search?author=L%20Yin), [Chen MX](https://www.citexs.com/Search?author=M%20X%20Chen), [Zeng TH](https://www.citexs.com/Search?author=T%20H%20Zeng), [Liu XM](https://www.citexs.com/Search?author=X%20M%20Liu), [Zhu F](https://www.citexs.com/Search?author=F%20Zhu), [Huang RQ](https://www.citexs.com/Search?author=R%20Q%20Huang). Improving probiotic spore yield using rice straw hydrolysate. Lett Appl Microbiol. 2021;72: 149–156[.](https://pubmed.ncbi.nlm.nih.gov/32939775) doi: [10.1111/lam.13387](https://doi.org/10.1111/lam.13387), [32939775](http://www.ncbi.nlm.nih.gov/pubmed/32939775).

10. Biao S, Weijing C, Yingqing Z, Jianjun L, Yinfang T, Fuming Z. Optimization of the spore-forming and high density culture conditions for *Bacillus coagulans*. J Hunan Agric Univ (Nat Sci). 2021;47: 171–179. doi: [10.13331/j.cnki.jhau.2021.02.008](https://doi.org/10.13331/j.cnki.jhau.2021.02.008).

11. Lina S, Xun J, Quanxing Z, Jinsong Z, Dongmei L. High cell density culture of *Bacillus* 13002. Food Ind Technol. 2017;38: 114–120. doi: [10.13386/j.issn1002-0306.2017.21.024](https://doi.org/10.13386/j.issn1002-0306.2017.21.024).

12. Peipei D, Xiangyan W, Yuanxiang L, Guoqin X, Haiyan X, Wei G, et al. Optimization of fermentation medium of *Bacillus coagulans*. China Brew. 2018;37: 28–32. doi: [10.11882/j.issn.0254-5071.2018.04.006](https://doi.org/10.11882/j.issn.0254-5071.2018.04.006).

13. Kaixiao L, Miao L, Shuaibiao L, Wanqiu Y, Hongfei M, Zhenzhen Z, et al. Optimized culture control for producing spores of *Bacillus coagulans*: 2019; 83–87. doi: [10.26914/c.cnkihy.2019.07106](https://doi.org/10.26914/c.cnkihy.2019.07106). [in Chinese].

14. Honghao D. Screening of *Bacillus coagulans* and high-density fermentation [D]. Hubei University of Technology. 2021. doi: [10.27131/d.cnki.ghugc.2021.000414](https://doi.org/10.27131/d.cnki.ghugc.2021.000414). Available from: https://kns.cnki.net/kcms2/article/abstract?v=3uoqIhG8C475KOm_zrgu4lQARvep2SAkueNJRSNVX-zc5TVHKmDNkqT7prPMUNk2tOj-RfOEu5WwKkK1RYb5QFX2VMfEToRx&uniplatform=NZKPT&src=copy

15. Yingying Q, Yanzhi L, Danling H, Nanxi L, Yi Y, Chengqun C. Screening and characterization of probiotics for fodder fermentation. Journal of Guangdong Industry Polytechnic. 2022;21: 1–7. doi: [10.13285/j.cnki.gdqgxb.2022.0001](https://doi.org/10.13285/j.cnki.gdqgxb.2022.0001)
