# Supplementary figures and images for: Isolation, functional evaluation, and fermentation process optimization of probiotic Bacillus coagulans
Source: PLoS One. 2023 Nov 3;18(11):e0286944. doi: 10.1371/journal.pone.0286944 (PMC10624278; doi:10.1371/journal.pone.0286944)

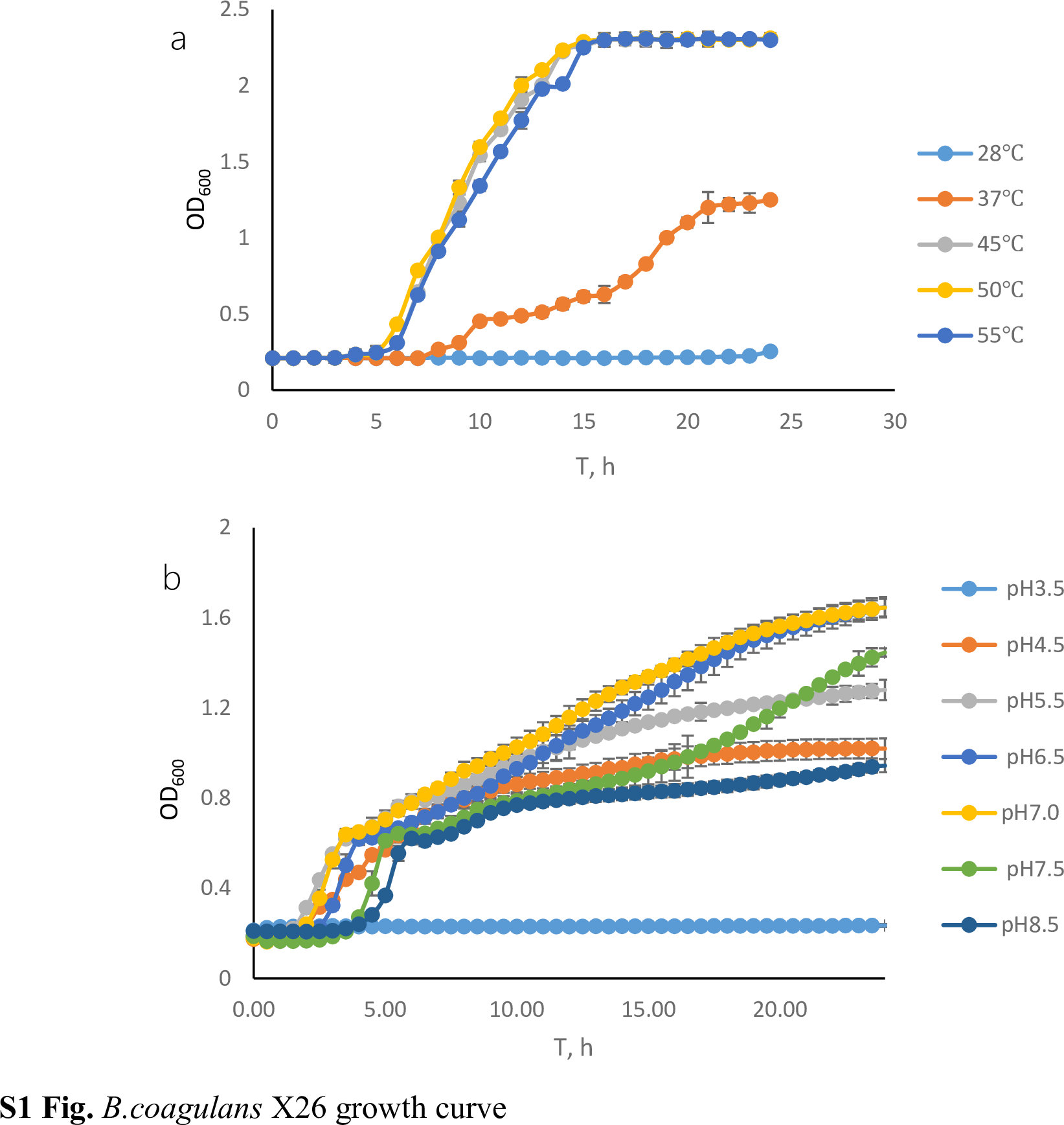

Supplement: S1 Fig — (TIF) [file pone.0286944.s006.tif]
